# Spatio-temporal feature extraction in sensory electroneurographic signals

**DOI:** 10.1098/rsta.2021.0268

**Published:** 2022-07-25

**Authors:** C. Silveira, R. N. Khushaba, E. Brunton, K. Nazarpour

**Affiliations:** ^1^ School of Engineering, Newcastle University, Newcastle upon Tyne NE1 7RU, UK; ^2^ Australian Center for Field Robotics, The University of Sydney, New South Wales 2006, Australia; ^3^ National Vision Research Institute, Australian College of Optometry, Carlton, Victoria 3053, Australia; ^4^ Department of Optometry and Vision Sciences, Faculty of Medicine, Dentistry and Health Sciences, University of Melbourne, Parkville, Victoria 3010, Australia; ^5^ Edinburgh Neuroprosthetics Laboratory, School of Informatics, University of Edinburgh, Edinburgh EH8 9AB, UK

**Keywords:** peripheral nerve recording, classification of sensory signals

## Abstract

The recording and analysis of peripheral neural signal can provide insight for various prosthetic and bioelectronics medicine applications. However, there are few studies that investigate how informative features can be extracted from population activity electroneurographic (ENG) signals. In this study, five feature extraction frameworks were implemented on sensory ENG datasets and their classification performance was compared. The datasets were collected in acute rat experiments where multi-channel nerve cuffs recorded from the sciatic nerve in response to proprioceptive stimulation of the hindlimb. A novel feature extraction framework, which incorporates spatio-temporal focus and dynamic time warping, achieved classification accuracies above 90% while keeping a low computational cost. This framework outperformed the remaining frameworks tested in this study and has improved the discrimination accuracy of the sensory signals. Thus, this study has extended the tools available to extract features from sensory population activity ENG signals.

This article is part of the theme issue ‘Advanced neurotechnologies: translating innovation for health and well-being’.

## 1. Introduction

Neuroprostheses have the potential to provide therapies in a wide range of fields. From established applications, such as spinal cord injury and prosthetic limbs [1–4], to recent advances in treating inflammatory diseases [5–7], there are numerous ways to benefit from interfacing with the human nervous system. In particular, interfaces with the peripheral nervous system (PNS) have been among the most successful in translating from research to clinical use [8–10]. Nevertheless, interfacing with the PNS comes with technological challenges and unanswered questions. At the interface level, some of the challenges include tissue damage and the foreign body response (FBR); hardware design and materials able to adapt, function and last in a biologically harsh environment; the trade-off between invasiveness and selectivity of recording/stimulation; and neural signal decoding challenges [10–12].

For most applications, and especially in devices that aim to provide sensory feedback in prosthetic limbs, there is need for bidirectional interfaces that can both record and stimulate peripheral nerves. However, most advances and clinical translation have been achieved in the stimulation field [2,3,13–16] while the long-term recording capabilities of the interfaces are lagging behind [17–19]. The low signal to noise ratio and the increasing distance from the recording electrode to the signal source due to encapsulation [12,19] make the task of long-term recording a difficult one. Nevertheless, the recording and consequent decoding of electroneurographic (ENG) signals is beneficial to equip neuroprostheses to provide more information to the user as well as to the system as a whole [17,20,21]. Electromyographic (EMG) signals have been the preferred source to record, decode and classify motor signals for prosthetic control thanks to, mostly, easier access [22,23] compared with ENG signals. Therefore, while numerous approaches have been proposed for EMG feature extraction and classification, less attention has been paid to extracting features from ENG signals. In particular, there are few studies that examine feature extraction on afferent nerve signals collected with extraneural interfaces [17,21,24].

Features are defined as the chosen transformation to represent the acquired signal [22] and are generally divided into three types: time-domain, frequency-domain and hybrid features. In EMG discrimination, it has been shown that the choice of features has more impact on classifier performance than the choice of the classifier itself [22,25–27]. Thus, it is important to investigate various feature extraction methods that are appropriate to extract information from ENG signals. Some of the approaches borrowed from EMG feature extraction and previously used in extraneurally recorded ENG signals include mean absolute value (MAV) [24,28,29], variance (VAR) [24], wavelength (WL) [24], autocorrelation-based features [24,30,31], autoregression coefficients [24] and Cepstral coefficients [24]. Time-domain features are popular due to their ease of implementation, effectiveness, and low computational cost [32]. Furthermore, Raspopovic *et al.* [24] reported that power-based features (MAV, VAR, WL and discrete Fourier transforms) yielded the highest classification accuracy results when discriminating different modalities of sensory ENG (proprioceptive, touch and nociceptive). Making the transition to the frequency-domain is valuable since these features provide a useful representation of the signal for classification. However, this transition also increases the computational cost and it can be disadvantageous in online processing scenarios. In previous studies [26,32,33], a set of power spectrum characteristics were extracted from EMG data without the conversion to the frequency-domain. With this approach, it was possible to benefit from the frequency-domain like information while staying in the time-domain and it achieved good results in EMG discrimination [32–34].

Other approaches to decode peripheral nerve activity recorded with extraneural interfaces include techniques such as velocity selective recording. This method allows to discriminate neural activity based on fibre conduction velocity. With this method, Metcalfe *et al.* [35] demonstrated that it was possible to discriminate, in real time, between afferent and efferent activity in the vagus nerve of pigs, as well as to extract firing rates of action potentials for a range of conduction velocities. Furthermore, other approaches involve the clustering of compound neural activity based on their waveform properties, such as shape and amplitude, to discriminate between different neural sensory groups [36]. The shape and amplitude of waveforms are affected by, among others, fibre size, propagation speed and fibre location relative to the recording electrode [36]. Moreover, a recent acute study by Koh *et al.* [37] used convolutional neural networks to discriminate sensory information recorded from the sciatic nerve of rats using a 56-channel nerve cuff. The study showed that high classification accuracies can be achieved using their CNN framework [37].

In this study, we sought to test and compare different feature extraction frameworks to discriminate proprioceptive neural data. The sensory signals were invoked by mechanical stimulation of the hindlimb of rats and recorded from the sciatic nerve using 16-channel nerve cuffs, in acute settings. The feature frameworks implemented here focused on taking into account the temporal and spatial aspects of the datasets, since the data was collected with multi-channel nerve cuffs, and also prioritized having a low computational cost. Furthermore, we apply a new framework that uses dynamic time warping (DTW) [38] in the feature extraction step, and that borrows concepts from deep learning to incorporate long-short-term memory. We believe that the frameworks tested here, or some of its strategies, can be useful for population activity ENG discrimination.

## 2 Methods

### (a) Data collection and pre-processing

The proprioceptive neural data was collected from four Sprague–Dawley rats over 4 days of acute in vivo experiments. All animal care and procedures were approved by the Animal Welfare and Ethical Review Board of Newcastle University. The full details of the experimental methods and surgical procedures can be found in [28,29]. In short, the 16-channel nerve cuffs were concentric nerve cuffs (Microprobes for Life Science: Gaithersburg, MD) measuring 4.25 mm in length and an inside diameter of 1.0 mm. The cuffs consisted of four rings, which were spaced by 0.75 mm. Each ring contained four platinum electrode contacts which were equally spaced within the ring. The cuffs were connected to a digital headstage (Cereplex-M32, Blackrock Microsystems), where the signal was digitized and bandpass filtered (300–7500 Hz), before being sent to the Cerebus Neural Signal Processor (Blackrock Microsystems). The sampling rate was 30 kHz and the signal was further bandpass filtered (250–5000 Hz). A stainless steel wire was placed in the skin, close to the site where the cuffs were implanted in the sciatic nerve, to serve as a reference for the recordings. The wire was held in place with tissue glue.

**Figure 1.**
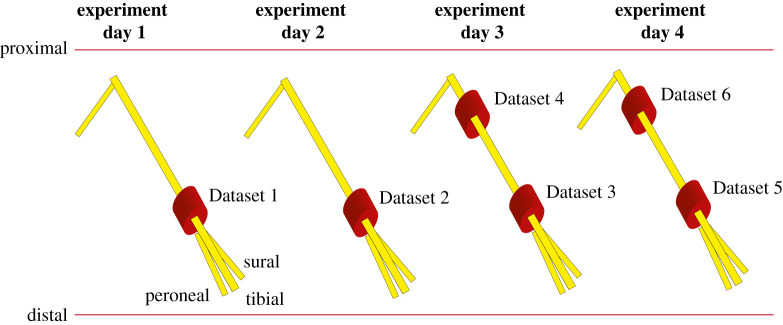
Schematic of the position of the 16-channel cuffs (red) on the sciatic nerve (yellow) on each experiment day. The distal position is closer to the site where the sciatic nerve divides into its peroneal, tibial and sural branches. Datasets 3 and 4 were collected simultaneously on experiment Day 3, and Datasets 5 and 6 were collected on experiment Day 4. (Online version in colour.)

Datasets 1 and 2 were collected on experiment Days 1 and 2, respectively, using a single 16-channel cuff implanted on the sciatic nerve on each experiment day. Datasets 3 and 4 were collected on experiment day 3, and Datasets 5 and 6 on experiment Day 4. On experiment Days 3 and 4, two 16-channel cuffs were implanted concurrently in the sciatic nerve, one distally and the other proximally to the site where the sciatic nerve starts to branch, figure 1. Mechanical stimulation was delivered automatically by using a standardized set-up where the rat’s toes were glued to a rod attached to a stepper motor, which was controlled by an Arduino [28,29]. The rat was placed on a sling so the hindlimb could be freely moved by the motor rod. The rat’s hindlimb was pseudo-randomly moved to six different angles ($-30^{\circ }$, $-20^{\circ }$, $-10^{\circ }$, $10^{\circ }$, $20^{\circ }$, and $30^{\circ }$) with respect to a neutral position ($0^{\circ }$). The neutral position corresponded to where the hindlimb would naturally rest when the rat was placed on the sling. Each dataset consisted of five blocks of proprioceptive stimulation, and each block delivered ten stimuli for each of the six angles, i.e. the six classes. Therefore, a total of 300 stimuli, each applied for 3 s, were collected per dataset. For every stimulus applied (stimulus ON), the hindlimb was subsequently moved to the neutral position for 3 s (stimulus OFF). For feature extraction, we were only interested in the steady-state response, i.e. when the hindlimb was not moving. Therefore, running observation windows (ROWs) were extracted from the 2.5 s of steady-state, removing 0.25 s of transient period at the start and end of stimulus application. This was done to ensure that motor movement related artefact would not contaminate the neural recordings.

The data were post-processed offline in Matlab and the first step consisted of performing spectral analysis to identify the signal’s energy and apply adequate filtering. In agreement with the literature, the energy peaks of the extracellular ENG signals were observed at frequencies lower than 2000 Hz [17,21,24,39]. Thus, a finite-impulse response bandpass filter (800–2200 Hz) was chosen to digitally filter the data [17,24]. The bandpass window accounted for reflexly evoked EMG contamination at frequencies below 800 Hz [24,31,40] and other unwanted signals such as high-frequency amplifier noise. The Neural Processing Matlab kit (NPMK, Blackrock Microsystems) was used to identify the stimuli application times using comments that were automatically written at the time of data collection to the file that also contained the neural data.

### (b) Feature extraction methods

**Figure 2.**
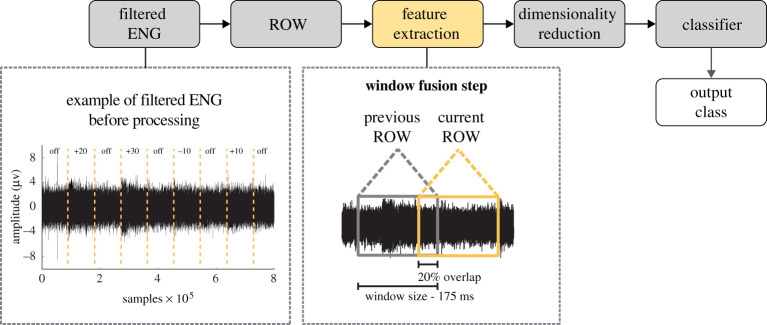
Sequence of the standard processing steps applied to the ENG data. An example of filtered proprioceptive data before processing is presented. A window fusion step was applied during the feature extraction step, meaning that the output features of each ROW were multiplied by the corresponding previous ROW, acting like a fusion step [33,34]. This step was applied in all feature extraction frameworks implemented in this study. The size of the ROW was 175 ms with 20% overlap. The ENG signal representation in the window fusion step is used for illustration purposes and is not scaled to represent the real window size of 175 ms. An orthogonal fuzzy neighbourhood discriminant analysis (OFNDA) was used to reduce the dimensionality of the data before feeding it to a linear discriminant analysis (LDA) classifier. (Online version in colour.)

The sequence of processing steps applied to the sensory data are summarized in figure 2. Firstly, a running observation window of 175 ms with a 20% window overlap was chosen after testing window sizes between 50 and 350 ms. There are no suggested values for ENG observation window sizes so it must be chosen empirically [17]. Secondly, five different feature extraction frameworks were tested on the ENG data, namely MAV; MAV and WL combined; time-domain descriptors (TDDs) [32–34]; temporal-spatial descriptors (TSDs) [26]; and spatio-temporal warping (STW) [41]. Differently from a standard processing sequence, a window fusion step was incorporated in all the feature extraction frameworks implemented in this study. The fusion step, proposed in [33,34], consisted of multiplying the output features from each running observation window with the features extracted from the previous observation window. This was inspired by Bayesian fusion and majority vote techniques that are normally applied after the classification step. However, the window fusion step is applied during the feature extraction step and, instead of multiplying probabilities after classification, the output features from two temporal windows are multiplied and normalized in every iteration. This approach incorporates temporal focus in feature extraction, and given the good results achieved in EMG classification [33,34] this approach was applied to the five feature extraction methods implemented in this study.

Table 1 presents some of the different feature extraction methods applied to the sensory data. The MAV and WL are some of most used standard features in EMG signal analysis [42] and have previously successfully been applied to ENG signals for classification of sensory data [24,28,29]. Features $f_{1}-f_{6}$ from table 1, are the TDD framework features derived using Fourier transform relations to extract power spectrum moments from a time window [33,34]. The TDD framework was proposed in [33,34] for EMG feature extraction, and here it was adapted to extract features from the ENG sensory data. The Fourier transform relations are based on Parseval’s theorem—the sum of the square of the function is equal to the sum of the square of its transform [43]

2.1 $$\sum_{j=0}^{N-1}|x[j]{|}^{2}=\frac{1}{N}\sum_{k=0}^{N-1}|X[k]X^{*}[k]|=\sum_{k=0}^{N-1}P[k],$$

where N is the number of temporal samples of the ENG signal; x[j] is the j-th sample of the signal; X[k] is the discrete Fourier transform (DFT) of x; k=0,1,…,N−1 is the frequency index; and P[k] is the result of the multiplication of X[k] by its conjugate $X^{*}[k]$ and is denoted as the phase excluded power spectrum [33,43]. From this theorem, it is possible to derive the root squared zero- ($m_{0}$), second- ($m_{2}$) and fourth ($m_{4}$)-order moments and from there features $f_{1}$ to $f_{6}$. Full details on how to derive each feature can be found in [33,34]. The TDD approach considers the temporal aspects of data by iteratively extracting the six features ($f_{1}-f_{6}$) from the current observation window as well as from the previous observation window, and from a nonlinear version of both the current and previous windows. The nonlinear version of the time windows corresponds to a logarithmically scaled version of the original signal: $\log{\left(x^{2}\right)}^{2}$, with x being the original ENG signal. The six features extracted from the original window $a=[a_{1},a_{2},\ldots ,a_{6}]$ and from the nonlinear version of the time window $b=[b_{1},b_{2},\ldots ,b_{6}]$ are correlated using a cosine similarity measure

2.2 $${\text{TDD SimilarityMeasure}}_{i}=\frac{-2a_{i}b_{i}}{a_{i}^{2}+b_{i}^{2}}\;\mathrm{for}\;i=1,\ldots ,6.$$

**Table 1.** List of the time-domain features applied to the ENG data. The TDD framework descriptors, features $f_{1}-f_{6}$, are introduced and derived in [33,34], and features $f_{7}-f_{8}$ are used in the TSD framework, presented in [26].

name	description	feature
mean absolute value	$\frac{1}{N}\sum_{j=0}^{N-1}|x_{j}|$	MAV
wavelength	$\sum_{j=1}^{N-1}|x_{j+1}-x_{j}|$	WL
root squared zero order moment	$\bar{m_{0}}={\left(\sum_{j=0}^{N-1}x{\left[j\right]}^{2}\right)}^{1/2};m_{0}=\frac{{\bar{m}}_{0}^{\lambda }}{\lambda };\lambda =0.1$	$f_{1}=\log(m_{0})$
root squared second order moment	$\bar{m_{2}}={{\left(\frac{1}{N}\sum_{j=0}^{N-1}(\Delta x[j]\right)}^{2})}^{1/2};m_{2}=\frac{{\bar{m}}_{2}^{\lambda }}{\lambda }$	$f_{2}=\log(m_{0}-m_{2})$
root squared fourth order moment	$\bar{m_{4}}={\left(\frac{1}{N}\sum_{j=0}^{N-1}{\left(\Delta^{2}x[j]\right)}^{2}\right)}^{1/2};m_{4}=\frac{{\bar{m}}_{4}^{\lambda }}{\lambda }$	$f_{3}=\log(m_{0}-m_{4})$
sparseness	$S=\frac{m_{0}}{\sqrt{m_{0}-m_{2}}\sqrt{m_{0}-m_{4}}}$	$f_{4}=\log(S)$
irregularity factor (zero crossings (ZC) divided by the number of peaks (NP))	$IF=\frac{ZC}{NP}=\sqrt{\frac{m_{2}^{2}}{m_{0}m_{4}}}$	$f_{5}=\log(IF)$
wavelength ratio	$WLR=\sum_{j=0}^{N-1}\frac{|\Delta x|}{|\Delta^{2}x|}$	$f_{6}=\log(WLR)$
coefficient of variation	$COV=\frac{\sqrt{(1/(N-1))\sum_{j=0}^{N-1}{\left(x[j]-\bar{x}\right)}^{2}}}{\left(1/N)\sum_{j=0}^{N-1}x[j\right]}$	$f_{7}=\log(COV)$
Teager–Kaiser energy operator	$\mathrm{TKEO}=\sum_{j=0}^{N-2}x{\left[j\right]}^{2}-x[j-1]x[j+1]$	$f_{8}=\log(TKEO)$

For each current observation window, the resulting six features of the combination of the original and nonlinear version of the signal (${\mathrm{SimilarityMeasure}}_{1}-{\mathrm{SimilarityMeasure}}_{6}$) were merged with those of the 3rd previous observation window. This accounts for the temporal information in the signal and acts as a fusion step. This approach results in higher correlation values when the two signals belong to the same class and in lower values otherwise. In [33], the use of a nth previous window instead of the previous window was suggested. Thus, here, the use of the 3rd previous window, in the TDD framework, was chosen empirically. In sum, the TDD framework consists of: extracting features ($f_{1}-f_{6}$ presented in table 1) from the original signal and from a nonlinear version of the signal; and correlating the output of the two (equation (2.2)). These two steps are applied to the current observation window, as well as to a previous observation window, the 3rd previous window in this case. The output features from these two windows are multiplied (window fusion) and this corresponds to what is fed to the classifier, after the process is repeated for the whole dataset.

Features $f_{7}-f_{8}$, table 1, were proposed in the TSD framework presented in [26], as an extension to the TDD framework [33,34]. Besides the incorporation of features $f_{7}-f_{8}$, the two algorithms differ since the TSD framework extracts the features ($f_{1}-f_{8}$) from each individual channel ($C_{x}$), as well as from all combinations of the difference between channels ($C_{x}-C_{y}$), and from a logarithmic scaled version of it. This adds a spatial component to the TSD framework since it looks at how the features extracted from the 16 channels in the cuff relate to each other. Another difference between the TDD and TSD algorithms is that the function used to correlate the features extracted from the signal’s current time window (x) and from its nonlinear version ($log{\left(x^{2}\right)}^{2}$) is a modification of equation (2.2)

2.3 $${\text{TSD Similarity Measure}}_{i}=\frac{a_{i}b_{i}}{\sqrt{(}\sum_{i=1}^{n}a_{i}^{2})+\sqrt{(}\sum_{i=1}^{n}b_{i}^{2})}\;\mathrm{for}\;i=1,\ldots ,n,$$

where n is the number of features extracted from each time window. The output features resulting from the correlation between the original and the nonlinear version of the signal (equation (2.3)), both from the within channel and the between-channel analysis, are then fed to the classifier.

**Figure 3.**
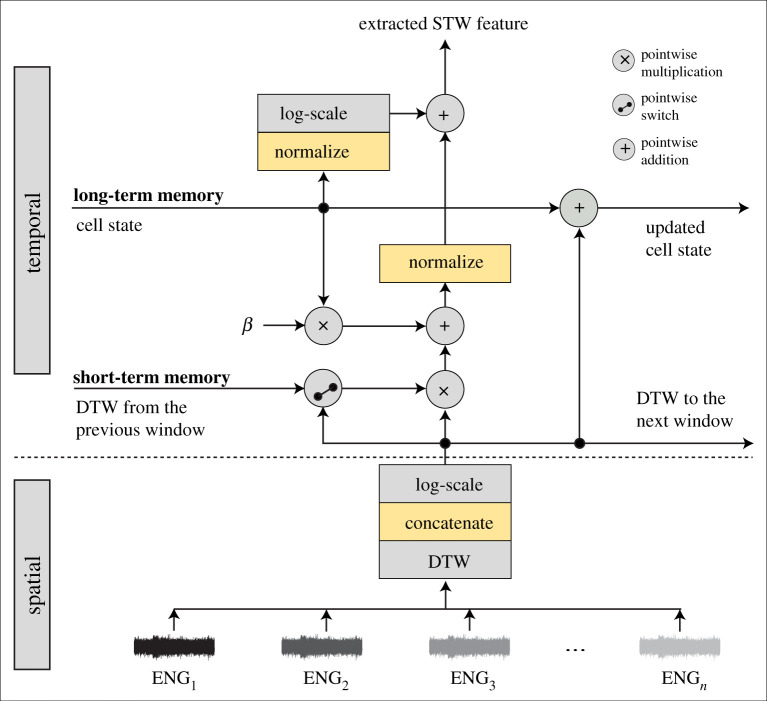
Diagram of the STW feature extraction framework. DTW is applied as a feature by measuring the similarities between two time windows of the ENG signals. This is done for all combinations of pair of recording channels, as well as to the first and second derivatives of the signal, adding spatial focus to the feature extraction. The output of the DTW is logarithmic scaled and added to the cell state in each iteration to act as a long-term memory component (cell state). Concurrently, the log-scaled DTW output is multiplied with the DTW output from the previous window, creating a short-term memory component (window fusion). The pointwise switch corresponds to the if clause that accounts for the first iteration of the code, when there is not yet a previous window. The information from the cell state is added to the output of the short-term memory after being multiplied by a weight factor (0.75≤β≤1.25). (Online version in colour.)

The STW framework, figure 3, is an adaptation of the TSD framework with two main differences. Firstly, instead of calculating the difference between every pair of channels to extract features from, the spatial component is applied with DTW [38,44]. DTW is applied as a feature and it outputs the similarity between two temporal sequences, in this case two time windows of ENG signals at a time. The similarities are highlighted because DTW looks for the best alignment between the time series with respect to a common time axis. The DTW is applied for every two time windows of all combinations of pair of channels. Additionally, the same procedure is applied to the first and second derivatives of each observation window. Thus, for every combination of two observation windows there are three sets of output values after the DTW feature is extracted. The second difference between the STW and TSD frameworks is that STW implements long-short-term memory in the feature extraction step. The short-term memory component corresponds to the window fusion step, obtained by multiplying the DTW output of the current window, after being logarithmic scaled, with the output from the previous observation window. The long-term memory component is implemented by having the equivalent to a cell state, or information highway, where information is taken from and added to in every iterative step. As shown in figure 3, the log-scaled DTW output is added to the cell state and, concurrently, the historic information from the cell state is also added to the DTW output after being multiplied by a weight parameter (0.75≤β≤1.25). Finally, the output is normalized and added to the log-scaled and normalized output of the cell state.

For each dataset, the number of extracted features with the STW framework is given by equation (2.4).

2.4 $$\text{number output features}=\frac{NC*(NC-1)}{2}*3,$$

where NC is the number of recording channels. Since the nerve cuffs used in this study have 16 recording channels there were 360 output features per dataset. For each time window, the DTW was extracted from every combination of pair of channels (NC∗(NC−1)) and this is divided by 2 since half of the combination of pair of channels is redundant information. Finally, the DTW feature is extracted from the original signal as well as from its first and second derivatives, thus the multiplication by 3 to have the total number of features extracted.

### (c) Dimensionality reduction and classification

An orthogonal fuzzy neighbourhood discriminant analysis (OFNDA) [45] was used to reduce the dimensionality of the feature space. This method, proposed in [45], was chosen thanks to its lower computational cost compared to other discriminant analysis methods such as uncorrelated linear discriminant analysis (ULDA) [45]. Furthermore, the OFNDA is a supervised dimensionality reduction method as it uses the class labels as one of the function inputs, enabling to retain its output predictive capabilities after the dimensionality transformation. The feature space was reduced to a maximum of six dimensions, which correspond to the number of proprioception classes. Further details on the OFNDA method can be found in [45].

The output features of each feature method were fed into a linear discriminant analysis (LDA) classifier and a 10-fold cross-validation was performed. The classification accuracy was calculated as the percentage of correctly classified instances resulting from the predictions of the 10-fold cross-validation.

For visualization purposes, the first two feature dimensions obtained with the STW, TSD and MAV & WL frameworks were scatter plotted.

### (d) Statistical analysis

Statistical analysis was performed to compare the classification scores obtained with the STW framework when using different window sizes (from 50 ms to 350 ms). Owing to the unknown distribution of the data, a non-parametric Kruskal Wallis test was used for the analysis with the Bonferroni method to compensate for multiple tests.

### (e) Computational time

The computational time required to extract features from one observation window was calculated for the five feature extraction frameworks implemented in this study. A window size of 175 ms, with sampling frequency of 30 kHz, and 10 channels were the parameters used to compare the computational time required for each of the frameworks to extract the features from one running observation window. The results are presented as the mean of 1000 runs. This analysis was done on a 2 GHz Quad-Core Intel Core i5 processor, 16 GB of RAM. Additionally, for the STW framework, the computational time per running observation window was calculated for window sizes ranging from 50 to 350 ms, in steps of 25 ms, and for different numbers of recording channels (3–16 channels).

## 3. Results

**Figure 4.**
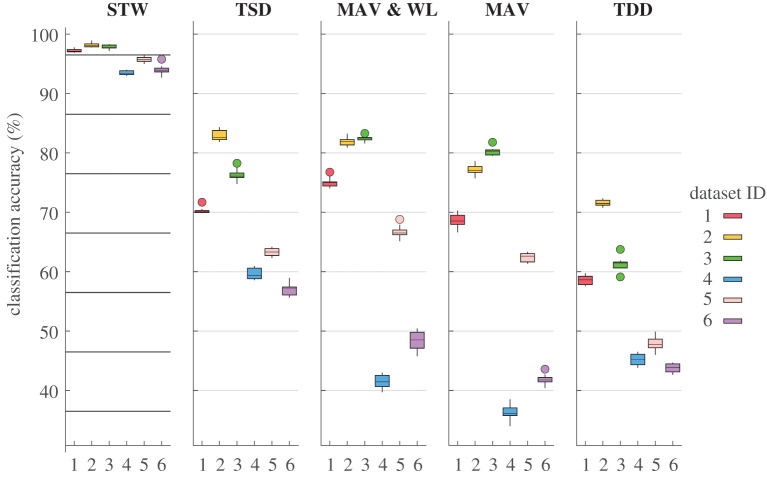
Classification accuracy results obtained with an LDA classifier (upon 10-fold cross-validation) using five different feature extraction frameworks: STW, TSD, MAV & WL, MAV and TDD on six datasets (Datasets 1–6). The size of the window was 175 ms. The information from the STW framework yielded the highest accuracy scores with an LDA classifier, with medians between 93.4 and 98.1%. The lowest accuracy results were observed for Datasets 4 and 6 and when using information from the MAV framework (median accuracies of 36.1% and 41.8%, respectively). The boxes are drawn between the 25th and 75th percentiles. The line indicates the median. The whiskers extend above and below the box to the most extreme data points that are within 1.5 times the interquartile range. The circles represent the outliers. (Online version in colour.)

The classification results obtained with an LDA classifier with 10-fold cross-validation are presented in figure 4. The six datasets correspond to the six cuffs acutely implanted in four different animals, figure 1. The STW framework performed the best across the six different datasets, in comparison with the other feature extraction frameworks. The median accuracies of correctly classified instances within the six datasets ranged from 93.4 to 98.1%. The TDD framework achieved the lowest median classification scores for Datasets 1–3 and 5, ranging between 47.7 and 71.5%. For Datasets 4 and 6, the MAV feature set obtained the lowest median scores (36.1% and 41.8%). Importantly, Datasets 4 and 6 correspond to data from proximally implanted cuffs for which the classification task is harder, which corroborates previous findings with the same dataset [28].

The median accuracy scores obtained for Datasets 1–3 and 5 with the MAV, MAV & WL, and TSD feature sets ranged from 62.5–80.1%, 66.7–82.8% and 70.4–86.4%, respectively. However, for Datasets 4 and 6, the median classification accuracies obtained were lower with the MAV (36.2% and 42.0%) and the MAV & WL (41.8% and 48.6%) frameworks than with the TSD (66.8% and 65.2%) framework, figure 4.

The supplementary material presents the confusion matrices of the STW framework to further complement the information presented in figure 4 and show which neighbouring classes resulted in misclassification.

**Figure 5.**
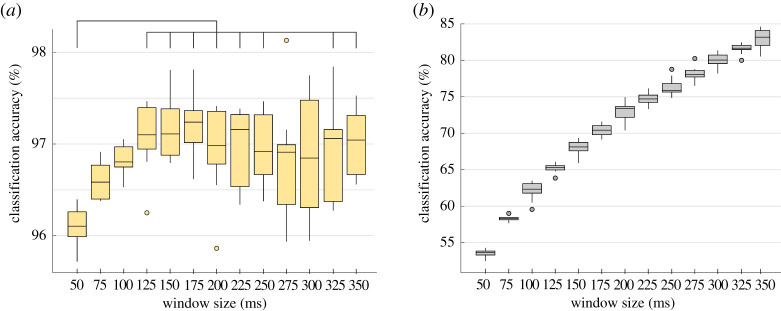
Classification accuracy obtained for Dataset 1 with (*a*) the STW feature set and (*b*) the TSD feature set, using a range of windows sized between 50 and 350 ms. The range of variation of classification accuracy scores was higher with the TSD framework (53.6–83.2%) than with the STW framework (96.1–97.2%). The head bars in (*a*) indicate statistically significant differences for the classification scores obtained with the STW framework. The boxes are drawn between the 25th and 75th percentiles. The line indicates the median. The whiskers extend above and below the box to the most extreme data points that are within 1.5 times the interquartile range. The circles represent the outliers. (Online version in colour.)

Figure 5 shows the classification accuracy results obtained for Dataset 1 using window sizes between 50 and 350 ms for the STW (figure 5*a*) and the TSD (figure 5*b*) frameworks. While the median results obtained with the STW framework varied between 96.1% and 97.2% of correctly classified instances, for the TSD feature set the results ranged from 53.6 to 83.2%. For the TSD framework, the larger the window size the higher the classification accuracy obtained. The bars in figure 5*a* show that there is a statistically significant difference in classification scores between using an observation window of 50 ms and the following window sizes: 125 ms ($p=9.23\times 10^{-4}$), 150 ms ($p=3.79\times 10^{-4}$), 175 ms ($p=1.38\times 10^{-4}$), 200 ms (p=0.0275), 225 ms (p=0.0085), 250 ms (p=0.042), 325 ms (p=0.0411) and 350 ms (p=0.0065).

**Figure 6.**
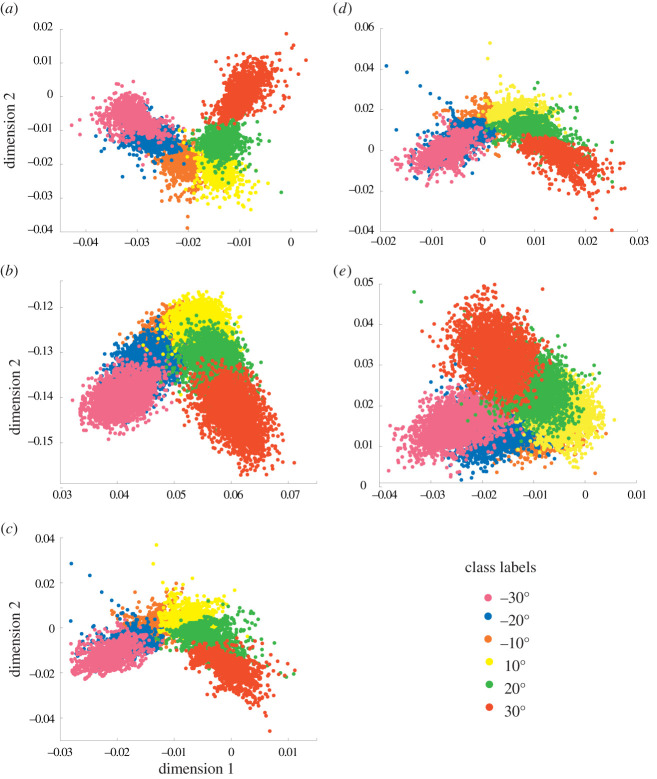
Visualization of the first two dimensions of Dataset 2 when applying the (*a*) STW, (*b*) TSD, (*c*) MAV & WL, (*d*) MAV and (*e*) TDD feature sets. (Online version in colour.)

Figure 6 presents the scatter plots of the first two dimensions, out of six, of Dataset 2 when using the five feature extraction frameworks implemented in this study (STW, TSD, MAV & WL, MAV and TDD). For the STW framework, figure 6*a*), the distribution of the data points appears to be tighter within the same class, and with less overlap.

**Figure 7.**
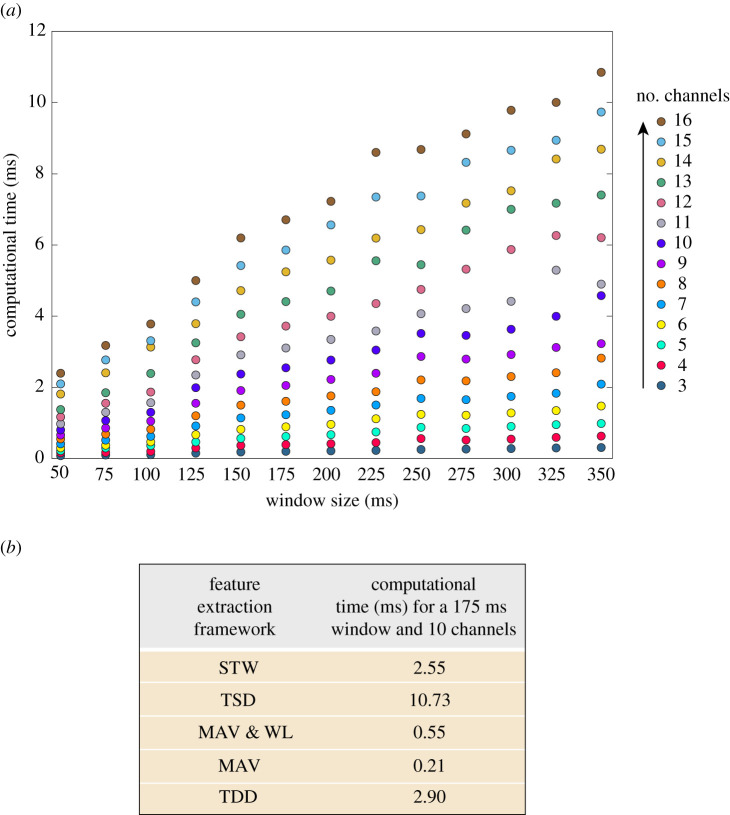
(*a*) Computational time (ms) versus window size (ms) for different numbers of recording channels (3–16) for the STW framework. (*b*) Computational time (ms) to extract the features from one observation window for the five feature extraction frameworks, when using a window of 175 ms and 10 recording channels. (Online version in colour.)

Figure 7*a* presents the computational time, in ms, required for the STW framework to extract features from one observation window for different window sizes (50–350 ms in steps of 25 ms) and for different numbers of recording channels (3–16 channels). The results show that the longer the window size, and the higher the number of recording channels, the higher the computational time to extract the STW features from the data. Figure 7*b* shows the computational time of the five feature extraction frameworks when using a window size of 175 ms and 10 recording channels. The MAV framework is the fastest to extract the features from the observation window, while the TSD framework is the slowest.

## 4. Discussion

In this study, five feature extraction methods were applied to sensory neural data collected with multi-channel cuff electrodes. The aim was to test and compare different sets of features that incorporate spatial and temporal focus in ENG feature extraction. The results show that the STW framework was the best performing method on the six datasets of sensory data and it could be used in ENG discrimination, especially when the computational costs must be kept low.

We believe that a few key factors contributed to the high classification accuracies obtained with the STW framework, making it the best-performing framework tested in this study. Firstly, it uses DTW as a feature to extract similarities between every pair of observation windows of all combinations of the sixteen recording channels. This has introduced spatial focus by taking advantage of the positioning of the 16 cuff contacts. This is especially relevant because different magnitudes of cumulative nerve activity can be observed at different locations along the nerve [46]. Thus, it was possible to relate the information acquired from the different electrode channels. Secondly, the window fusion step, i.e. the multiplication of the output features of the current observation window with the previous window, introduced temporal focus in the feature extraction process. Furthermore, the incorporation of a long-term memory component has contributed to taking the historic information into account. We believe that the combination of all of these factors resulted in the improvement of the feature extraction process of the sensory ENG datasets compared to the remaining frameworks tested. Nevertheless, the MAV, MAV & WL and the TDD frameworks did not perform as well as the STW framework even with the implementation of temporal and spatial focus. In these frameworks, the lowest results were seen for Datasets 4 and 6, which correspond to the cuffs that were implanted proximally in the sciatic nerve. As reported in [28], at a proximal location the different fibres of the sciatic nerve are less divided than at a distal location, where the cuff is closer to the peroneal and tibial branches division. Even so, the STW framework could still discriminate the different proprioceptive angles in Datasets 4 and 6 with high levels of accuracy while remaining in the time-domain.

The design of the STW algorithm was inspired by deep learning methods [41]. Like the STW algorithm, deep learning techniques such as convolutional neural networks (CNNs) and long-short-term memory (LSTM) can exploit the spatial correlations between the data and the long-term, nonlinear dynamics of time-series data, respectively. However, these methods also need long training times, large amounts of training data, and generate large amounts of model parameters. The advantage of the STW framework is that it enables the incorporation of the benefits of some deep learning concepts while keeping a low computational cost. The results of the computational time analysis showed that the processing speed of the STW framework was slower compared to the MAV and MAV&WL frameworks. This was expected given the higher complexity of the STW framework. Nevertheless, the computational time of the STW framework is desirable for real-time applications and showed a faster processing speed compared to the TSD and TDD frameworks.

The choice of the observation window size for this study was done empirically after implementing windows ranging from 50 ms to 350 ms. We present the results of the impact of the window size on the classification scores for the two best performing feature sets: the STW and the TSD frameworks. The STW framework showed high classification scores with both short and long running observation windows but the highest median values were normally achieved with windows of 125 ms, 150 ms or 175 ms. A statistically significant difference in classification performance was seen between windows of 50 ms and the following sizes: 150 ms, 175 ms, 200 ms, 225 ms, 250 ms, 325 ms and 350 ms. By contrast, with the TSD framework the window size had more impact on the classification scores. For example, in dataset 1, figure 5, the median classification accuracy of a 10-fold cross-validation varied 29.5 percentage points with the TSD, and only 1.14 percentage points with the STW framework across the different window sizes. Thus, for the TSD method a larger observation window resulted in higher classification accuracies. This is because with a larger window size there is better representation of the signals’ characteristics in the time and frequency domains. However, when translating algorithms for real-time applications, such as bidirectional prosthetic limbs, there is a limit on how large the observation window can be before a delay can be perceived by the user. Window sizes of 100–250 ms have shown to provide a good trade off between classification performance and delay in myoelectric control [47,48]. On the other hand, more recent abstract myoelectric control studies have successfully used larger running observation windows [49]. In ENG processing, there is no standard value for the size of the running observation window. Nevertheless, a study by Raspopovic *et al.* [24] found that 100 ms windows provided the best performance in sensory ENG discrimination and larger windows saw a decline in performance. In this study, the STW framework did not have a statistically significant decline in performance when using large windows (greater than 300 ms) compared to 100 ms windows. Therefore, this adds to the strength of the STW framework. This framework provides flexibility in choosing an appropriate window size for the intended application, as evidenced by the high scores achieved across the range of window sizes tested in this study. It is important to consider, however, that the lower the window size the higher the run time of the algorithm, as seen in figure 7.

The feature frameworks implemented here are normally employed in EMG feature extraction and there are no studies investigating the applicability of these feature sets in ENG processing. Understandably, given its non-invasive nature, surface EMG signals are considerably easier to collect than peripheral nerve signals and there is a relatively direct link between EMG control signals and the user’s movement intention [50]. Additionally, ENG recordings are affected by the movement and potential displacement of the neural interface in the long-term, as it usually implanted next to highly mobile joints. Besides, there are numerous sources of noise that can contaminate ENG recordings such as EMG signals from the surrounding muscles, transient artefacts, oscillatory or time-varying noise [10,21,50,51]. However, despite the many challenges surrounding peripheral nerve signal collection, these recordings are likely to be useful tools in diverse applications of neuroprostheses. For example, ENG recordings can be useful in decoding more complex tasks for prosthetic control that surface EMG electrodes alone might not be able to [17,51]. Moreover, in case of amputation, there might not always be residual muscles available for recording [17,21]. Other applications of ENG recording and decoding include feedback control, or the acquisition of biomarkers from, for instance, the vagus and the carotid sinus nerve to aid in the treatment of diseases such as rheumatoid arthritis [5,7,10]. Furthermore, recording and decoding motor and sensory signals from the peripheral nervous system plays an important role in expanding our knowledge of neural structures [20].

Intraneural electrodes, such as the TIME [11], have shown very promising results in recording and discriminating neural signals to be used in bidirectional prosthetic devices [52,53]. Furthermore, these electrodes have been tested in chronic animal studies to investigate their stability over time [54] as well as in mid-term human studies [10,13,15]. However, intraneural electrodes are susceptible to increased FBR compared with extraneural, less invasive, interfaces [10,17]. While it has been shown that intraneural electrodes remain functional and selective in long-term animal stimulation studies, encapsulation of the electrodes within the fascicles still occurs [55–57], making long-term recording of neural signals challenging. For these reasons, it is important to consider the application before choosing the type of neural interface. Extraneural interfaces can still be a preferred option depending on the aim of the study or the clinical application. In stimulation studies, the flat interface nerve electrode (FINE) has proved to be stable and selective over time [2,55,58,59]. However, there is less progress in the clinical translation of long-term recording and discrimination of population ENG signals [60,61]. Nevertheless, source extraction algorithms have been developed to extract the neural signals of interest from mixed nerves using FINEs both in benchtop [62] and chronic settings [63,64]. It is important to continue to explore strategies to record and discriminate neural signals to improve neuroprostheses and the information provided to the user. The recording and processing of compound neural activity will be advantageous when the use of extraneural interfaces is more appropriate.

## 5. Conclusion

Different feature extraction methods were applied to sensory neural data to discriminate six classes of proprioceptive stimulation. The data were recorded from the sciatic nerve of rats in response to mechanical stimulation of the hindlimb. Multi-channel nerve cuffs were used to collect the neural signals. The feature frameworks implemented temporal and spatial focus in the feature extraction process, taking advantage of the sixteen contacts distributed along the nerve. The STW framework was the best performing for all the datasets tested in this study. This framework uses DTW as a feature to find similarities between the signals and incorporates long-short-term memory while keeping a low computational cost. We believe this algorithm can be used as a tool for population activity ENG feature extraction.

## Data Availability

Data supporting this publication is openly available under an ‘Open Data Commons Open Database License’. Additional metadata are available at: http://dx.doi.org/10.17634/141353-3. Please contact Newcastle Research Data Service at rdm@ncl.ac.uk for access instructions. Electronic supplementary material is available online [65].
